# Culture as both a risk and protective factor for vicarious traumatisation in nurses working with refugees: a literature review

**DOI:** 10.1177/17449871221085863

**Published:** 2022-07-08

**Authors:** Hannah Dodds, David J Hunter

**Affiliations:** Staff Nurse, Nursing & Health Care School, 3526 University of Glasgow, Glasgow, UK; Lecturer, Nursing & Health Care School, 3526 University of Glasgow, Glasgow, UK

**Keywords:** culture, literature review, refugees, vicarious resilience, vicarious traumatisation

## Abstract

**Background:**

There are an estimated 25.9 million refugees worldwide, who require health services while living in host countries. To effectively treat refugee patients, nurses must document their history which requires hearing about their traumatic journeys. Listening to trauma has been shown to cause vicarious traumatisation.

**Aims:**

To identify the risk and protective factors involved in the development of vicarious traumatisation.

**Methods:**

After searching four databases, nine studies were selected for review. Key words ‘vicarious trauma’, ‘refugee’ and ‘nurse’ formed the search. Articles were appraised using the Critical Appraisal Skills Program and Mixed Methods Appraisal Tool.

**Results:**

Vicarious traumatisation is common amongst nurses working with refugees. Aspects of culture formed the principal risk and protective factors. Differences between cultures made for strained communication while similarities created better understanding. Some cultures provided more resilience than others. Vicarious resilience, a feeling of personal growth resulting from hearing about and helping patients overcome trauma, was also highlighted. The development of vicarious resilience was a protective factor.

**Conclusions:**

Further investigation into how to minimise risk and establish protective factors is required. Some coping recommendations include personal reflection, comprehensive training and better access to counselling.

## Introduction

Following the Vietnam War, there was an emergence of trauma literature studying the impact of traumatic events on individuals (Figley, 1995). In 1980, Post-traumatic Stress Disorder (PTSD) was first recognised as a medical condition which started an in-depth investigation into people’s experiences of trauma (Figley, 1995). From this, there developed an interest in those psychologically and physically affected by hearing of people’s trauma rather than personally experiencing it. Their clinical presentation was principally referred to as compassion fatigue (Figley, 1995).

Instances of compassion fatigue amongst nurses are well documented, particularly in emergency departments, intensive care units and oncology centres (Hooper et al., 2010). These fields represent some of the most emotive areas in healthcare due to the life and death scenarios they give rise to and intensity of emotions involved. For nurses working in more extreme and less studied areas, such as in warzones and relief work, the most appropriate term for what they experience is vicarious traumatisation (Dunkley and Whelan, 2006). This is due to the traumatic descriptions given by those they are helping to treat which is out with the norm of what a nurse generally encounters.

There are currently an estimated 25.9 million refugees worldwide (United Nations High Commissioner for Refugees (UNHCR), 2019), in 2018 alone there were 1.1 million new refugees (Amnesty International, 2019). To put that figure into perspective, one person is displaced from their home due to conflict or persecution every 2 seconds (United Nations (UN), 2019). People do not choose to become refugees; they are forced to leave their homes due to war, violence or natural disaster. As a result, people are compelled to seek a safer and more secure life (Action Against Hunger, 2019).

The worldwide nature, and number, of refugees means they encompass people from many distinct cultures, beliefs, religions and with varying health states. It has been noted that many refugees arrive in host countries with undiagnosed or untreated health problems including lack of vaccination, nutritional deficiencies and infectious diseases, which creates a disparity between the physical and mental health of refugees and the host population and which is exacerbated by language, cultural barriers and inadequate health literacy (Suurmond et al., 2013). A successful first contact with the healthcare system is vital to ensure continued involvement in refugees' own wellbeing to promote better ongoing health (Suurmond et al., 2013). As a result, nurses are in a prime position to be able to help refugees with both their physical and mental health.

Suurmond et al. (2013) highlight that nurses themselves have identified that a discussion must be had about the patient’s journey to uncover any past medical history, hidden illnesses and about family situations to determine a patient’s level of resilience and therefore their ability to care for their own health needs. This inevitably leads to stories of the trauma they have experienced which nurses have been given no guidance on how to handle (Suurmond et al., 2013). In addition, there is insufficient time to support patients through trauma so nurses may feel they have inadequately helped ease worries and despair (Dummit and Honein-AbouHaidar, 2019). These circumstances can leave them vulnerable to developing vicarious traumatisation which impacts upon their physical and mental wellbeing. Nurses suffering from vicarious traumatisation can struggle to provide the best care to their patients due to decreased compassion reserves, anxiety and depression (Hooper et al., 2010). Nurses are known to be integral to the healthcare system, without them normal functioning is impossible, and patient outcomes and care are compromised (Gatchel, 2018). It is therefore important to minimise the negative effects on nurses which could cause them to leave the profession. To gain a better understanding of the intricacies surrounding the development of vicarious traumatisation, the risk and protective factors resulting in its’ development will be discussed.

## Terminology

Within this paper there will be a focus on refugees and not asylum seekers. As defined by the United Nations (2019), all refugees have been asylum seekers but not all asylum seekers will be granted residence in the country they apply for. As a result, by focussing on refugees the study encompasses everyone who has sought international protection, from the first day they arrive through all subsequent years. Refugees also represent a larger proportion of forcibly displaced people worldwide. According to the UNHCR (2019) only 4.94% of displaced people are asylum seekers compared to 36.58% refugees. The remaining 58.33% are made up of internally displaced people who have not crossed a border to find safety but are moving within their home country (UNHCR, 2019).

It has been noted that terms such as compassion fatigue, burnout, vicarious traumatisation and secondary traumatic stress have been used synonymously by some academics while others have chosen to define them individually (Kjellenberg et al., 2014; Phelps et al., 2009 and Puvimanasenghi et al., 2015). Burnout is defined as physical and emotional exhaustion caused by the cumulative impact of stress (Kjellenberg et al., 2014; Phelps et al., 2009). In comparison, compassion fatigue encompasses those who experience too great a demand on their emotions leading them to feel a sense of shared suffering and sorrow. Secondary traumatic stress is more specific to those working with survivors of trauma and incorporates elements of fear due to the nature of what they are hearing (Kjellenberg et al., 2014). Vicarious traumatisation encompasses a cognitive framework adjustment, creating a gradual inner change in response to stimuli that affects a person’s identity, world view and spirituality (Kjellenberg et al., 2014; Phelps et al., 2009). This means those who suffer from it struggle to maintain healthy personal relationships and everyday functioning in a world they no longer understand. According to Dunkley and Whelan (2006), vicarious traumatisation is the most appropriate term to describe working with refugees as it relates specifically to trauma. Therefore, vicarious traumatisation will be used to encompass all the above terms. Due to their similarity, it is possible to combine them, as academics have done before, under the heading which is most appropriate for the population being studied.

## Methodology

A literature search was carried out from September to November 2019. Four databases were used: Medline, Web of Science, PsychINFO and the Cumulative Index of Nursing and Allied Health Literature (CINAHL). All four databases were chosen because they are specific to healthcare. The PICo acronym (population, interest and context) was used to determine the key words needed to ensure the most effective literature search (Pollock and Berge, 2018). These were then combined with Medical Subject Headings (meSH) to find the initial article pool. Truncated symbols were placed at the end of words to include variations and ensure full coverage of the topic. The key words were vicarious trauma*, refugee* and nurs*. Terms were combined using Boolean operator ‘and’ which ensured all search entries were included in the articles found. Most articles, however, were found through citation searching, which Fain (2015) sees as the highest priority when researching.

Inclusion and exclusion criteria were set to provide focus to the search and ensure the most relevant material was uncovered. Both were devised from a review of the question as advised by Khan et al. (2003). Inclusion and exclusion criteria were decided based on population, language and cultural limitations, outcome variables and methodological quality (Meline, 2006). They were then evaluated on their relevance and acceptability (Robey and Dalebout, 1998). The cultural competence of papers was also included in the inclusion and exclusion criteria to ensure sensitive handling of information and appropriate techniques were used when collecting research (Pelzang and Hutchinson, 2018). Studies were excluded from analysis if they met at least one exclusion criteria, were poor in quality or had insufficient methodology (Meline, 2006). The criteria can be found in Table 1. A date range was not set due to there being only a small number of studies as a result of it being an emerging field. Therefore, restricting the search to only contemporary articles would limit findings.

**Table 1.** Inclusion and exclusion criteria – this table highlights the inclusion and exclusion criteria which were applied when undertaking the literature search.

Inclusion criteria	Exclusion criteria
Written or translated into English	Non-English papers
Clinicians working with refugees as focus of article with mention of nurses included	Studies focussing on therapists as their interaction is expected to involve in-depth discussions of past traumatic events
Demonstration of cultural competence towards participants	Papers that only focused on the experience of refugees and not the clinicians
—	Articles about burnout rather than terms under the vicarious traumatisation bracket.

**Figure 1.**
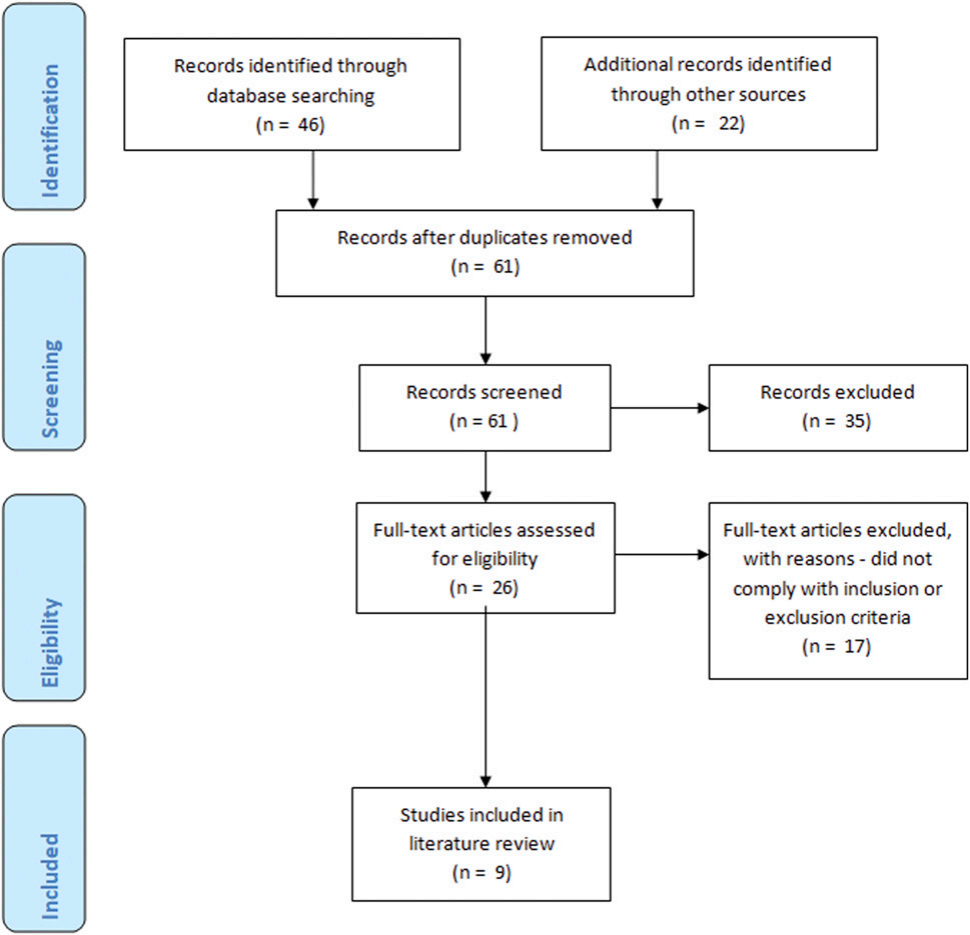
PRIMSA diagram – this figure illustrates the stages of the selection process which allowed the number of papers included in the review to be screened and reduced to those included in the review. Initially, 68 papers were identified. This was reduced to 9 to be included in the review.

The selected papers were apprised using the Critical Appraisal Skills Program (CASP) tool and Mixed Methods Appraisal Tool (MMAT) to ensure validity to the study and consistency in appraisal of articles. The CASP specialises in healthcare research and focuses on evaluating qualitative research which is included in all but one of the chosen articles (CASP, 2019). The MMAT tool is specific to mixed methods research of which there are three papers within this review. All articles are from peer reviewed journals meaning the content within has been assessed for validity by other academics (Manchikanti et al., 2015). The papers are from around the world and focus on different refugee populations. It was decided that having a variety of locations was acceptable as only a few in-depth studies on the subject have been undertaken so far. This means there is variation in experience between groups, but the overarching themes and findings are the same. The process of selecting papers is shown in a PRISMA diagram (Figure 1), an evidence based and globally recognised method of displaying literature search methods (PRISMA, 2019).

## Results

The search terms were input to the databases resulting in 46 articles being found. Once duplicates were removed there were 39 sources to review. An analysis of their titles and abstracts using the inclusion and exclusion criteria eliminated 35 articles leaving just four. The remaining articles were read in full to ascertain usefulness before a citation search of their bibliographies was undertaken. Twenty-two more articles were identified and had their abstracts screened, five were found to be relevant to this study. This left a total of nine papers to be used in the literature review. Table 2 provides an overview of the key features of the included papers.

**Table 2.** Evidence table – this table provides an overview of the key features of the nine papers included in the review.

Study	Aim/Purpose	Study design	Sampling and sample size	Data collection method	Key findings
Posselt et al., (2019)	Impact of working therapeutically with refugees on psychological wellbeing of clinicians	Mixed methods. Online survey- impact of work and protective factors. Open ended questions for qualitative data. Approx 30 min to complete. Collection between June ‘16- March ‘17	Purposive. 18 + working therapeutically with refugees in Australia/Australian run refugee processing centres. Recruited by contacting agencies who provide service. Approval gained from management and email sent with link to survey to all eligible staff. 50 took party, 47 majority, 41 complete	Demographic questions. Three scales: Depression, anxiety and stress scale (DASS-21), ProQOL and open ended questions	Stress increases in line with population norms, same with deprivation. High levels of compassion satisfaction. Low levels of burnout and STS. Males had more anxiety than females, full time more than part time. Less time working in trauma meant more anxiety. Government impact is substantial – feeling of hopelessness. Personal identity challenged. Government impact bigger than hearing stories of trauma. Support team – managers/colleagues determine level of coping. Allowed perspective and meaning making
Lusk and Terrazas, 2015	Impact on caregivers and professionals who give care to people severely traumatised by displacement	Mixed methods. 2x structured interviews + 2x self-administered measures (secondary traumatic stress scale + ProQOL 5). Participants select location/do on their own. Time period not given	Purposive. 18+ residing in EL Paso currently providing care/support to migrants who have experienced trauma. 11 professionals took part	Secondary traumatic stress scale + ProQO. Interviews transcribed verbatim, coded, validated through data	0% at least occasional numbness/trouble sleeping/intrusive thoughts/easily annoyed. All reported thinking about clients when they didn’t want to. 90% got satisfaction from helping people. All proud of work, 80% happy with line of work About 10% scored in severe/high range on STSS.
Kjellenberg et al., (2014)	How helpers working with severely traumatised individuals are affected by their work	Quantitative. Internet questionnaire (for ease) went to 12 centres working with war/torture survivor centres in Sweden	Purposive. 69 people working with war/torture survivors participated. Many professionals including nurses	Post-traumatic growth inventory (Swedish translation), ProQOL (not translated), standard acute stress reaction questionnaire – shortened to what was relevant to the questions being asked, traumatic experience checklist (translated) – 10 selected form possible 25 and opened ended questions at end for other comments. Fear and resignation towards human evil – self developed for the study (piloted before study and adjusted)	Hours per week had no effect but years in the job increased compassion fatigue and post-traumatic growth. Previous trauma caused impairment and post-traumatic growth. Workers who themselves had been refugees experienced higher levels of post-traumatic growth. No depersonalisation/unpainted function declared but responses suggest affected 1/4. 33% had change in attitude to human evil – more afraid and resigned towards it
Dumit and Honein-AbouHaidar 2019	To explore Lebanese nurses perspectives on the impact of Syrian refugee care on nurses working in hospitals and primary healthcare centres in Lebanon	Qualitative. Structured in-depth interviews. Recruited through ‘Order of Nurses’ in Lebanon. Interviewer had no connection to interviewees. Lasted 20 min on average. Between 1 May-1 June 2017, additionally December 2018	Purposive. 6 primary healthcare nurses and 6 nursing directors working in areas with high concentration of Syrian refugees	Structured in-depth interviews. Thematic reflections made. Sample saturation after 10th interview	2 main themes emerged. Profile of Syrian refugees and determinants of health/impact of Syrian refugees on nurses and beyond. Shift from acute to chronic over the years (first arrival vs. residence). Poor baseline health – no vaccines, poor living conditions, don’t know how to access healthcare. Gradually developed compassion reserve. Unprepared for the change but used it as a learning opportunity
Guhan and Leibling-Kalifani 2011	The experiences of staff working with refugees in the UK. The impact and range of psychical effects	Mixed method design. In-depth interviews with people working at one refugee centre (analysed with grounded theory) and brief self-reported questionnaire. Took place at the centre. Took around 1 h to interview then questionnaire. Opportunity for debrief after	Purposive. 12 people working in the refugee centre	Semi-structured in-depth interview and ProQOL questionnaire	Moderate level of satisfaction for workers with 25% very high satisfaction. Most not at risk of burnout but 25% high risk – could be their mood on that day. Compassion fatigue above 75th percentile. Client expectation often too high – unrealistic demands. Pleasure of seeing change happen and being a part of it. Emotional rollercoaster. Personal growth noted – changes to personality and character. Learn about other cultures. Changing world view – less trusting of people. Same culture/religion helped – include personal experience. Worried they seem incapable of they ask for help
Puvemanasenghi et el. 2015	Exploring the experiences of healthcare workers caring for refugees in south Australia	Qualitative. Semi- structured with open ended questions. Lasted 1–2 h. Recorded and transcribed. Offered to be interviewed in groups or alone	Purposive. 26 semi-structured face to face interviews with service providers working with refugees in Adelaide. 1/2 part of minority group, for example, migrant/recent refugee	Semi-structured interviews. Most individual but 2 in group. Transcripts repeatedly reviewed	Post-traumatic growth implied. Vicarious traumatisation doesn’t developing cultural awareness to help understanding. Language differences a barrier to providing care. Stigma in some cultures about mental health. Frustration with Australian government – systematic barriers for refugees
Griffiths et al. 2003	Identifying the skills, knowledge and support nurses require when working with refugees in reception centres in Australia	Qualitative. All nurse/midwives working in safe havens over 14 months invited to take part – 14 responses. 2 focus groups of 7, semi- structured format with interview schedule. Trigger questions relating to prior experience, nature of work, short/long term priorities, knowledge and skills required, nature of emotional support. Issues relating to returning to normal posts. 60–90 min in durations. Recorded and transcribed	Purposive. 13 nurses and 1 clerk in 2 focus groups plus two in-depth interviews with nursing managers	Thematic analysis of information gained from focus group and interview transcripts by multidisciplinary research team. Correlated into themes	All need help re: building patient identity and dignity. Includes treating health conditions with a social dimension. Experience prejudice from friends/family against refugee culture. Moral distress when they have to make decisions about health for reparations. Coping mechanisms included detachment/focusing on positive elements. Reasons to do it again – professional development of clinical skills – important but other elements impact on their ability to care as well. Coping with stress was treated as a personal matter, should be occupational
Barrington and Shakespeare-Finch, 2013	To examine the lived experience of people working on a daily basis with survivors of torture and trauma who had sought refuge in Australia	Qualitative. Semi-structured interview 45–60 min. All staff told of project via email with information sheet attached. Audio recorded and transcribed with personal information removed	Snowball. 17 trained clinicians, 13 administrative/management staff from Queensland Programme of Assistance to Survivors of Torture and Trauma (QPASTT)	Semi-structured interview. Unique interaction between interviewer and interviewer shaped course of interview and topics discussed	Cannot have growth without experiencing trauma. Traumatic stories primary cause not trauma. To reduce psychological distress they can adjust beliefs to incorporate traumatic stories and make meaning of experiences. 100% employed coping mechanisms – meditation, mindfulness, healthy eating, work/life balance, speaking to friends/family – colleague better, more understanding. Proud of work/growth of patient. Change to life philosophy, interpersonal relationships. Vicarious traumatisation a response in early stages of work. Due to the initial shattering of beliefs – stress reduced because of meaning making. Potential impact on personal and organisational level. Make sense of trauma, not bury the head
Barrington and Shakespeare-Finch, 2013	To examine whether hearing about trauma can be highly distressing and simultaneously provide an opportunity for positive personal growth	Qualitative. Longitudinal qualitative design with 2 semi-structured interviews a year apart. Follow up interview 30–45 min. Aim of first was to examine lived experiences of people working with survivors of refugee related trauma, focus on how they made sense of stories and if there were positive outcomes. Second – any change in experience	Snowball. Year 1–17 trained clinicians, administrative/managerial staff from QPASTT. Year 2–9 frontline clinicians, 3 administrative had left organisation	Semi-structured interviews. Longitudinal qualitative study. 4 open ended questions and several prompt questions	Consistent themes- vicarious trauma, challenges for clinicians, rewards for clinicians, coping strategies. Differences in themes: changes to policy impacting on how they do their work

## Discussion

Vicarious traumatisation has been shown to be present to some extent in every research study analysed in this review. The evidence suggests vicarious traumatisation has significant clinical implications for the individual and wider health systems. As a result, it is beneficial to consider the risk and protective factors associated with its development. Other themes were identified during the literature review however the scope of this paper did not allow for them to be discussed in further detail.

### Risk factors

Several studies (Guhan Liebling-Kalifani, 2011; Lusk and Terrazas, 2015; Puvimanasenghi et al., 2015) within the literature review investigated the effects of culture on the client and caregiver. They revealed that healthcare professionals from a different culture to their patient can sometimes fail to contemplate cultural implications and therefore provide inappropriate treatment (Marsella, 2010). For example, western councillors treating survivors of an earthquake in Taiwan put a heavy emphasis on talking through problems which was ineffective as Taiwanese culture generally does not support sharing feelings and emotions (Marsella, 2010). A lack of understanding between patients and caregivers could have an impact on the professional due to a breakdown in communication and progress leaving the caregiver feeling inadequate and as if they had failed. This can facilitate the development of vicarious traumatisation.

Marsella (2010) discusses how individuals with a culture which has historically endured traumatic circumstances through oppression, war and disaster are likely to have constant trauma with little resilience or hope of recovery. Therefore, caregivers who are from cultures which have been oppressed, such as those from Latin America working in the United States, are more at risk of developing vicarious traumatisation since their resilience is low and suffering trauma is almost expected.

### Culture as a protective factor

In the study conducted by Lusk and Terrazas (2015), there is an emphasis on cultural similarities and their impact. They state that a major protective factor for the Hispanic professionals working with Mexican refugees was that they shared cultural beliefs that helped to overcome trauma. These included kinship and familial support systems, faith and cultural conceptions. Similarly, Guhan and Leibling-Kalifani (2011) found that healthcare professionals with the same culture or religion as the refugee population were more protected against the effects of vicarious traumatisation. According to Southwick et al. (2011) culture determines how an individual copes with trauma and that the methods for doing so vary widely between cultures. Therefore, shared culture provides greater understanding between the two and allows for solutions specifically tailored towards the common culture to be employed by both parties. Almost 70% of caregivers working with the Mexican refugees in the Lusk and Terrazas (2015) study were also of Hispanic origin and all spoke fluent Spanish. The results showed that 90% of the caregivers reported satisfaction with their job. In comparison, a sample from the UK showed over 65% had a different cultural identity to the refugees they were caring for. Within the sample there were only moderate levels of vicarious resilience but on average workers were at risk of developing vicarious traumatisation, with 42% possibly already experiencing it (Guhan and Liebling-Kalifani, 2011). This suggests that having a shared culture can help protect healthcare professionals from being as afflicted by vicarious traumatisation.

Ethnocultural variations exist in presentations of PTSD (Marsella, 2010). Marsella (2010) suggests that culture constructs our reality and therefore influences the way we interact with and view the world. Many professionals believe almost every element of trauma-related mental health illness is influenced by culture which creates a template for how an individual experiences it (Marsella, 2010). These effects would be the same for victims of vicarious traumatisation. Some cultures appear to be more resilient to trauma than others, in particular Latino cultures because of the reasons mentioned above (Marsella, 2010). Consequently, caregivers from certain ethnocultural backgrounds are more protected from vicarious traumatisation due to the way culture has constructed their reality.

### Shared experience as a protective factor

It has been identified that caregivers who themselves have a migrant past, either directly or as a recent descendant, are better equipped to assist refugees (Lusk and Terrazas, 2015). They have experienced accumulative stress and adjustment to a dominant culture themselves and can therefore empathise and provide tried and tested solutions to problems non-migrants may not consider (Lusk and Terrazas, 2015). Those who had been refugees before becoming involved in supporting them reported higher levels of vicarious resilience than those who had not experienced the reality of being a refugee (Kjellenberg et al., 2014). Kjellenberg et al. (2014) link this with finding meaning in trauma. They note that it has positive psychological implications which protect against the negative impact of trauma. Those who have experienced trauma before have higher levels of post-traumatic growth through meaning making as they have been able to reflect upon their experiences and chose a field of work where they can help others do the same (Helgeson et al., 2006). Therefore, previous trauma and the subsequent ability to find meaning within it is a protective factor against, not vicarious trauma itself as it is needed to allow growth, but the everyday effects it can have.

### Vicarious resilience

The term vicarious resilience encompasses the positive outcomes of working with trauma survivors (Puvimanasenghi et al., 2015). It emerges once individuals can make sense of trauma and can result in positive cognitive changes such as altered views of themselves and of the world (Barrington and Shakespeare-Finch, 2014). These changes include gained knowledge and insight into the world due to an enriched perspective which bolsters their appreciation for their life in comparison to the refugees they interact with (Posselt et al., 2019). Every participant in the Barrington and Shakespeare-Finch (2014) research reported philosophical transformation relating to the world and the impact their job can have. These positive consequences have been shown to reduce symptoms of vicarious traumatisation (Posselt et al., 2019).

Vicarious traumatisation causes stress, among other symptoms. Cultural resilience has been identified as an effective stress management technique (Lusk and Terrazas, 2015). This is due to the amount people are supported within their cultural support system which influences an individual’s ability to handle adversity (Lusk and Terrazas, 2015). In Latino culture, caregiving is regarded as a duty and not a burden (Lusk and Terrezas, 2015). Therefore, it is unsurprising that caregivers of refugees who are Latino experienced high levels of vicarious resilience in the Lusk and Terrazas (2015) study. In comparison, Anglo-American caregivers felt their culture had a detrimental effect on the situation and their feelings (Lusk and Terrazas, 2015). Many even felt more removed from their values as, since they were part of the dominant culture, they could see the harmful effects it was having. They felt detached from their own upbringing but in many cases, this prompted them to develop a new cultural identity for themselves which provided them with some protection for combating vicarious traumatisation (Lusk and Terrazas, 2015).

According to Barrington and Shakespeare-Finch (2014) vicarious trauma is the natural initial response to hearing about refugee trauma followed by the development of vicarious resilience through meaning making of the situation and subsequent personal growth. They argue that preoccupation with vicarious traumatisation obstructs the reality of an overall positive outcome that manifests as vicarious post-traumatic growth. The opposite, however, could also be argued. Some studies (Kjellenberg et al., 2014; Posselt et al., 2019) showed lower levels of vicarious traumatisation symptoms in their self-reported questionnaire research than expected. This could be due to the overall effect of vicarious resilience. Within the transcripts and data collected it is clear everyone was negatively affected by hearing about refugee trauma. It can therefore be hypothesised that because of the positive effects of post-traumatic growth and vicarious resilience, caregivers do not necessarily self-report vicarious trauma. Instead, they subconsciously focus on the positives until encouraged to consider their feelings more closely.

### Limitations

Only four databases were used to conduct the literature search, allowing for potential research papers to be missed if they were not on the selected databases. Additionally, the review was only conducted by one researcher, as an undergraduate nursing dissertation, which leaves room for unintentional bias and a lack of alternative perspectives.

## Conclusion

The aim of this review was to determine the risk and protective factors surrounding the development of vicarious traumatisation amongst those working with refugees. Culture has emerged as the key element in both features. Although culture has been shown through numerous studies to be a risk factor for vicarious traumatisation, its crucial role in encouraging vicarious resilience helps to counteract the fact it can expose people to more trauma.

As mentioned, this study has its limitations however it creates an overview of current literature in the area which can be used as a base for further study into the subject with the aim of finding a solution to minimising vicarious traumatisation.

### Recommendations

As stated by Posselt et al. (2019), the wellbeing of clinicians has a direct impact on patients. To allow nurses to continue to be emotionally available and responsive to refugees, certain measures need to be implemented to reduce vicarious traumatisation.

Many of the risk and protective factors uncovered from the literature review are non-changeable as they are engrained in culture and history. Since recruitment based upon cultural identity is unethical, alternative risk and protective factors need to be explored.

### Recommendations for practice

The following changes to practice are recommended to reduce the instances of vicarious trauma amongst nurses working with refugees:

• Greater availability of counselling for nurses working with refugees. This would facilitate discussion around the subject of trauma to relieve feelings of isolation and to provide helpful coping mechanisms.
• Facilitation of time for reflection that can enhance the instances of meaning making which has been shown to encourage post-traumatic growth as part of vicarious resilience to overcome the effects of vicarious traumatisation.
• Provide comprehensive training to nurses prior to entering a position which will require interaction with refugee patients. If they are made aware of the signs for developing vicarious traumatisation they can take responsibility for their own mental health and seek help when necessary.
• Ongoing monitoring of all nursing staff working with refugees using a combination of the questionnaires such as the ProQOL (Stamm, 2010) to determine those at a higher risk of, or those who have already developed, vicarious traumatisation.

### Recommendation for future research

The following areas require investigation to learn how to reduce the instances of vicarious traumatisation amongst nurses working with refugees:

• Further research into changeable risk factors to discover how to reduce the development of vicarious traumatisation. This includes risk and protective factors out with culture that can be applied to the whole workforce.

**Key points for policy, practice and/or research**

- Vicarious traumatisation affects almost every healthcare professional who interacts extensively with refugees and consequently requires investigation to minimise the impact on caregivers and their patients.
- Culture is both a risk and protective factor for vicarious traumatisation and can therefore potentially be manipulated into promoting resilience over traumatisation with further research.
- Vicarious resilience has been shown to have a positive impact for almost everyone and so ongoing investigations into combating vicarious traumatisation should focus around encouraging quicker and more sustainable development of vicarious resilience.
- More training on the subject to provide awareness and enable caregivers to recognise its development in themselves is an important next step in its treatment.
